# E2A selectively regulates TGF‐β–induced apoptosis in KRAS‐mutant non‐small cell lung cancer

**DOI:** 10.1002/1878-0261.70236

**Published:** 2026-03-17

**Authors:** Sergei Chuikov, Shiva Krishna Katkam, Zhefan Wang, Venkateshwar G. Keshamouni

**Affiliations:** ^1^ Division of Pulmonary and Critical Care Medicine, Department of Internal Medicine University of Michigan Ann Arbor MI USA; ^2^ Program in Chemical Biology University of Michigan Ann Arbor MI USA; ^3^ Rogel Cancer Center, University of Michigan Medical School Ann Arbor MI USA; ^4^ LTC Charles S. Kettles VA Medical Center Ann Arbor MI USA

**Keywords:** bHLH transcription factor, E12/E47, KRAS, lung adenocarcinoma, radiation therapy, SMAD3

## Abstract

Transforming growth factor‐β (TGF‐β) regulates epithelial homeostasis by inducing growth arrest and apoptosis during early carcinogenesis; however, these tumor‐suppressive functions are frequently lost in advanced nonsmall cell lung cancer (NSCLC) despite intact signaling. We identify the transcription factor E2A as a critical mediator of resistance to TGF‐β‐induced apoptosis in mutant KRAS–driven NSCLC. TGF‐β induces E2A expression in a SMAD3‐dependent manner in NSCLC cells harboring mutant KRAS, but not in those with wild‐type KRAS. Silencing E2A restores TGF‐β‐induced apoptosis in mutant KRAS cell lines without affecting epithelial–mesenchymal transition. E2A depletion promotes mitochondrial apoptosis through mitochondrial outer membrane permeabilization, caspase‐3 activation, and regulation of BCL‐2 family and inhibitor‐of‐apoptosis proteins. In contrast, wild‐type KRAS NSCLC cells fail to upregulate E2A in response to TGF‐β and remain resistant to apoptosis following E2A silencing. Knockdown of mutant KRAS abrogates the pro‐apoptotic effects of E2A silencing, establishing KRAS dependency. E2A silencing enhances radiation‐induced growth inhibition, likely through increased sensitivity to TGF‐β signaling. E2A is overexpressed in lung adenocarcinoma and is significantly elevated in tumors harboring mutant KRAS. These findings identify E2A as a context‐specific suppressor of TGF‐β‐mediated apoptosis and a potential therapeutic target in mutant KRAS NSCLC.

## Graphical abstract

Ability to induce apoptosis by TGF‐β is frequently lost in advanced lung adenocarcinoma despite intact TGF‐β signaling. We identify E2A as a mutant KRAS–dependent mediator of resistance to TGF‐β–induced apoptosis. TGF‐β induces E2A via SMAD3 in mutant KRAS cells, and E2A silencing restores apoptosis and enhances radiation response in cell lines harboring mutant KRAS.

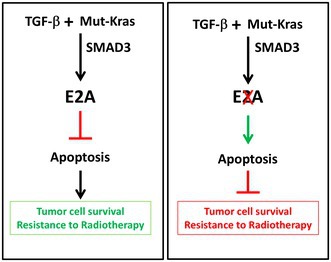

**Abbreviations**
ALL: Acute lymphoblastic leukemia
ANOVA: Analysis of variance
ATCC: American Type Culture Collection
BIRC: Baculoviral IAP repeat containing (inhibitor of apoptosis proteins)
BSRB: Biomedical Science Research Building
CAGA‐luc: SMAD‐responsive luciferase reporter construct
CO₂: Carbon dioxide
DMEM: Dulbecco's modified Eagle medium
E2A: E12/E47 transcription factor (encoded by TCF3)
ECL: Enhanced chemiluminescence
ELISA: Enzyme‐linked immunosorbent assay
EMT: Epithelial‐to‐mesenchymal transition
FN1: Fibronectin
GAPDH: Glyceraldehyde 3‐phosphate dehydrogenase
Gy: Gray (unit of radiation dose)
HRP: Horseradish peroxidase
IAP: Inhibitor of apoptosis protein
JC‐1: MitoProbe JC‐1 dye
KRAS: Kirsten rat sarcoma viral oncogene homolog
LUAD: Lung adenocarcinoma
MAPK: Mitogen‐activated protein kinase
MOMP: Mitochondrial outer membrane permeabilization
mRNA: Messenger RNA
mTOR: Mammalian target of rapamycin
NF‐κB: Nuclear factor kappa B
NSCLC: Nonsmall cell lung cancer
PBS: Phosphate‐buffered saline
PI3K: Phosphoinositide 3‐kinase
PVDF: Polyvinylidene difluoride
qPCR: Quantitative polymerase chain reaction
RIPA: Radioimmunoprecipitation assay buffer
RNA: Ribonucleic acid
RRID: Research Resource Identifier
SD: Standard deviation
SEM: Standard error of the mean
siRNA: Small interfering RNA
SMAD: Mothers against decapentaplegic homolog
TCF3: Transcription factor 3
TCGA: The Cancer Genome Atlas
TGF‐β: Transforming growth factor beta
TGF‐β1: Transforming growth factor beta 1
TGFβRII: Transforming growth factor beta receptor II
UMCCC: University of Michigan Comprehensive Cancer Center
WT: Wild‐type
XIAP: X‐linked inhibitor of apoptosis protein

## 1 Introduction

Transforming Growth Factor‐β (TGF‐β) is a multifunctional cytokine that regulates a plethora of biological processes including cell growth, differentiation, and apoptosis [1]. TGF‐β elicits its effects by binding to type‐II and type‐I serine/threonine kinase receptors on the cell surface, triggering the assembly of a hetero‐tetrameric receptor complex. Type‐II receptor kinase transactivates type‐I receptors, which in turn phosphorylate receptor SMADs, SMAD2, and SMAD3. Phosphorylated SMAD2/3 engaged in a complex with SMAD4 translocate into the nucleus, interacting with different co‐activators or corepressors to regulate target gene transcription through specific DNA binding motifs [2, 3]. In addition to SMAD‐dependent signaling, TGF‐β also activates SMAD‐independent signaling pathways, which include mitogen‐activated protein kinases (MAPKs), Rho‐like GTPases, Phosphoinositide‐3 kinase (PI3K), mTOR, and NF‐kB pathways [4].

In cancer, TGF‐β acts as a tumor suppressor in early stages and as a tumor promoter in the late stage of tumor progression [5]. As part of its tumor suppressor function, TGF‐β is capable of inducing apoptosis in premalignant and malignant cells [6]. TGF‐β activates apoptosis by directly modulating the apoptotic proteins or indirectly by inducing the expression of proteins that regulate pro‐apoptotic signaling pathways [7]. During tumor progression, some cancer cells acquire resistance to the tumor suppressive effects of TGF‐β due to loss or mutations in one of the components of the TGF‐β signaling pathway, including TGF‐β receptor II (TGFβRII), SMAD2 and SMAD4 [8]. Interestingly, the majority of cancer cells have intact and functional TGF‐β signaling, and yet they become refractive to the tumor suppressive effects of TGF‐β. TGF‐β now switches to a tumor promoter, favoring tumor progression and metastasis by inducing epithelial–mesenchymal transition (EMT), enhancing angiogenesis, imparting drug resistance, and evading host immune surveillance [9, 10]. The ability of TGF‐β to exert its tumor suppressive functions in cancer cells may be hindered by the induction of molecules that serve as molecular switches between tumor suppressive and tumor promoting pathways of TGF‐β. Identification and characterization of such molecules may provide therapeutic opportunities to restore tumor‐suppressive functions of TGF‐β.

Transcription factor E2A (E12/E47), encoded by the gene *TCF3*, regulates gene expression by binding with either corepressors or co‐activators on the target gene promoters [11]. E2A plays a critical role in lymphocyte development [12, 13], but not much is known about its role in other cell types including epithelial cells. E2A overexpression as a fusion protein (E2A‐PBX1, E2A‐HLF and E2A‐FB1) is frequently associated with ALL [14]. However, no E2A fusion proteins were reported in solid tumors. Very little is known about the role of E2A in the oncogenesis of non‐lymphoid organs including lung. E2A was originally identified as an E‐cadherin repressor and promotes EMT leading to increased migration and invasion [15], but its impact on TGF‐β‐induced apoptosis has not been defined. Here, we identify E2A as a molecular switch selectively suppressing TGF‐induced apoptosis in mutant KRAS NSCLC. Importantly, E2A upregulation in response to TGF‐β and its functional role in apoptosis suppression and radiation sensitivity are restricted to mutant KRAS NSCLC lines, but had no effect on the TGF‐β‐induced EMT. Consistently, E2A is overexpressed in tumors compared to normal lung tissue and elevated in tumors mutant KRAS compared to tumors with wild‐type KRAS in patients with lung adenocarcinoma.

## 2 Materials and methods

### 2.1 Cell culture

Human lung cancer cell lines A549 (Cat# CRM‐CCL‐185, RRID:CVCL_0023), H358 (Cat# CRL‐5807, RRID:CVCL_1559), H522 (Cat# CRL‐5810, RRID:CVCL_1567), H23 (Cat# CRL‐5800, RRID:CVCL_1547), H460 (Cat# HTB‐177, RRID:CVCL_0459), and H2170 (Cat# CRL‐5928, RRID:CVCL_1535) were obtained from the American Type Culture Collection (ATCC, Manassas, VA, USA). The HCC78 lung adenocarcinoma cell line (Cat# 302156, RRID:CVCL_2061) was obtained from Cell Lines Service LLC (Sioux Falls, SD, USA). All cell lines were cultured in RPMI‐1640 medium with L‐glutamine (Cat# SH30027.01, Cytiva, Marlborough, MA, USA), supplemented with 10% heat‐inactivated fetal bovine serum (Cat# A52568‐01, Gibco, Grand Island, NY, USA), and 1% penicillin/streptomycin (Cat# SV30010, Cytiva). Cells were maintained in a humidified incubator at 37 °C with 5% CO_2_. Cell lines have been authenticated in the past three years; authentication was performed by ATCC via short tandem repeat (STR) analysis. Cell lines were tested for mycoplasma contamination, all experiments were performed with mycoplasma‐free cells. For EMT experiments, cells were seeded in 6‐well plates at 30–40% confluency (50 000 cells/well) and grown in complete medium. Cells were serum‐starved for 24 h and then treated with 5 ng/mL TGF‐β1 (Cat# A1123, PeproTech, Cranbury, NJ, USA) for 72 h. Where indicated, morphological changes were monitored in real time using the IncuCyte live‐cell imaging system (Essen BioScience, Ann Arbor, MI, USA).

### 2.2 Live‐cell time‐lapse imaging

A549 cells were serum‐starved for 24 h and stimulated with TGF‐β1(5 ng/mL) for 72 h. Cells were continuously monitored using the IncuCyte live‐cell imaging system located within a standard incubator. Images were captured every 2 h and compiled into .mp4 format and Caspase‐positive cells were quantified using the IncuCyte software.

### 2.3 Western blot analysis

Following treatment, cells were washed with PBS and lysed in RIPA buffer supplemented with NaF, Na_3_VO_4_, and protease inhibitors. Protein samples (20 μg) were separated on SDS/PAGE and transferred to PVDF membranes. Membranes were probed with the following primary antibodies from Cell Signaling Technologies (Beverly, MA, USA): E2A (Cat# 4865, RRID:AB_10560512), GAPDH (Cat# 97166, RRID:AB_2756824), fibronectin (FN1; Cat# 26836, RRID:AB_2924220), E‐cadherin (Cat# 3195, RRID:AB_2291471), N‐cadherin (Cat# 4061, RRID:AB_10694647), phospho‐SMAD2 (Cat# 8828, RRID:AB_2631089), and total SMAD2 (Cat# 5339, RRID:AB_10626777), phospho‐SMAD3 (C25A9) (Cat# 9520, RRID:AB_2193207), and total SMAD3 (C67H9) (Cat# 9523, RRID:AB_2193182). Vimentin (Cat# SAB4300676, RRID:AB_11130230) was obtained from Sigma‐Aldrich (St. Louis, MO, USA). After overnight incubation with the primary antibody at 4 °C, membranes were incubated with HRP‐conjugated secondary antibodies and visualized using Clarity Western ECL substrate (Cat# 1705061, Bio‐Rad, Hercules, CA, USA). Protein expression was quantified by measuring the pixel intensity of protein bands in Fiji v1.54p (RRID:SCR_002285). Band intensities were normalized to the corresponding GAPDH loading control, or to total SMAD2 or SMAD3 for phospho‐SMAD2 or phospho‐SMAD3. The normalized protein expression values are shown above each respective band.

### 2.4 siRNA transfection

SMARTpool siRNAs targeting E2A (Cat# M‐009384‐00‐0005), KRAS (Cat# L‐005069‐00‐0010), SMAD2 (Cat# L‐003561‐00‐0010), and SMAD3 (Cat# L‐020067‐00‐0010) were purchased from Dharmacon (Lafayette, CO, USA). A scrambled siRNA (Cat# D‐001810‐10‐05) served as a control. Cells were seeded at 30–40% confluency (50 000 cells/well) and transfected using Lipofectamine 2000 (Cat# 11668027) in Opti‐MEM (Cat# 31985062; Thermo Fisher Scientific, Waltham, MA, USA). After 6 h, cells were switched to complete RPMI‐1640 medium and allowed to recover overnight before further treatments.

### 2.5 Quantitative PCR analysis

Total RNA was extracted using the E.Z.N.A. Total RNA Kit I (Cat# R6834‐00S, Omega Bio‐tek, Norcross, GA, USA). Primers for E2A, GAPDH, Bad, Bak1, Bax, Caspase‐3, ‐6, ‐9, BIRC3, BIRC8, XIAP were purchased from IDT Inc. (Coralville, IA, USA). qPCR was performed on an ABI Prism system (Applied Biosystems, Carlsbad, CA, USA), with gene expression normalized to GAPDH. Thermal cycling conditions were: 94 °C for 30 s, 50 °C for 1 min, 68 °C for 45 s, repeated for 40 cycles. Fold changes were calculated relative to control. Expression of apoptotic genes (Bad, Bak1, Bax, Caspase‐3, ‐6, ‐9, BIRC3, BIRC8, XIAP) was analyzed using a pathway‐specific qPCR SuperArray (SABiosciences/QIAGEN, Germantown, MD, USA) following the manufacturer's protocol and confirmed by qPCR.

### 2.6 Analysis of clinical datasets

E2A mRNA expression in LUAD tumors vs. normal tissues and in wild‐type vs. mutant KRAS tumors was analyzed using TCGA‐LUAD data (https://portal.gdc.cancer.gov/projects/tcga‐luad) via the R statistical software. Graphs were generated using Prism v10 (RRID:SCR_002798, GraphPad, Boston, MA, USA).

### 2.7 TGF‐β signaling assays

For signaling assays, cells were serum‐starved overnight and stimulated with TGF‐β1 (5 ng/mL). SMAD2 and SMAD3 phosphorylation were analyzed by western blot using phospho‐SMAD2 and phospho‐SMAD3 antibodies. SMAD‐mediated transcriptional activity was assessed using a SMAD‐responsive luciferase reporter (CAGA × 3‐luc), transfected with Lipofectamine 2000, and measured using the Dual‐Luciferase Assay System (Cat# E1910, Promega, Fitchburg, WI, USA).

### 2.8 Cell proliferation and apoptosis assays

Cell proliferation was measured using the CellTiter‐Glo luminescent assay (Cat# G7570, Promega) 72 h post‐treatment. Cell death was evaluated by trypan blue exclusion. Apoptosis was assessed by Annexin V/7‐AAD staining (Cat# 640922, BioLegend, San Diego, CA, USA) followed by flow cytometry (Attune NxT, Thermo Fisher Scientific). Data were analyzed using FlowJo v10.8 (RRID:SCR_008520, BD, Ashland, OR, USA). Caspase‐3 activation was detected with NucView 488 substrate (Cat# 30029, Biotium Inc., Fremont, CA, USA) and analyzed by fluorescence microscopy and flow cytometry.

### 2.9 Mitochondrial outer membrane permeability assay

MOMP was evaluated using JC‐1 dye (Cat# T3168, Thermo Fisher Scientific). Cells were stained for 30 min at 37 °C and analyzed by flow cytometry. A decrease in the red:green fluorescence ratio indicated loss of MOMP.

### 2.10 ELISA for TGF‐β1 secretion

A549 cells were irradiated (0–10 Gy; cesium‐137 source, BSRB, University of Michigan). Conditioned media were collected 24 h postirradiation, and TGF‐β1 levels were measured using the human TGF‐β1 Quantikine ELISA Kit (Cat# DLAP00, R&D Systems, Minneapolis, MN, USA), following acid activation of latent TGF‐β.

### 2.11 Radiation and clonogenic survival assay

Cells transfected with siRNA were irradiated at 5 Gy and plated for colony formation. After 10 days, colonies were stained with crystal violet and counted. Colony formation efficiency was calculated relative to non‐irradiated controls.

### 2.12 Statistical analysis

All data are presented as mean ± SD. Statistical analyses were performed using prism v10.0 (RRID:SCR_002798, GraphPad). Comparisons between groups were made using one‐way ANOVA or Student's *t*‐test. *P* values < 0.05 were considered statistically significant.

## 3 Results

### 3.1 Lung cancer cells are resistant to TGF‐β‐induced apoptosis

**Fig. 1:**
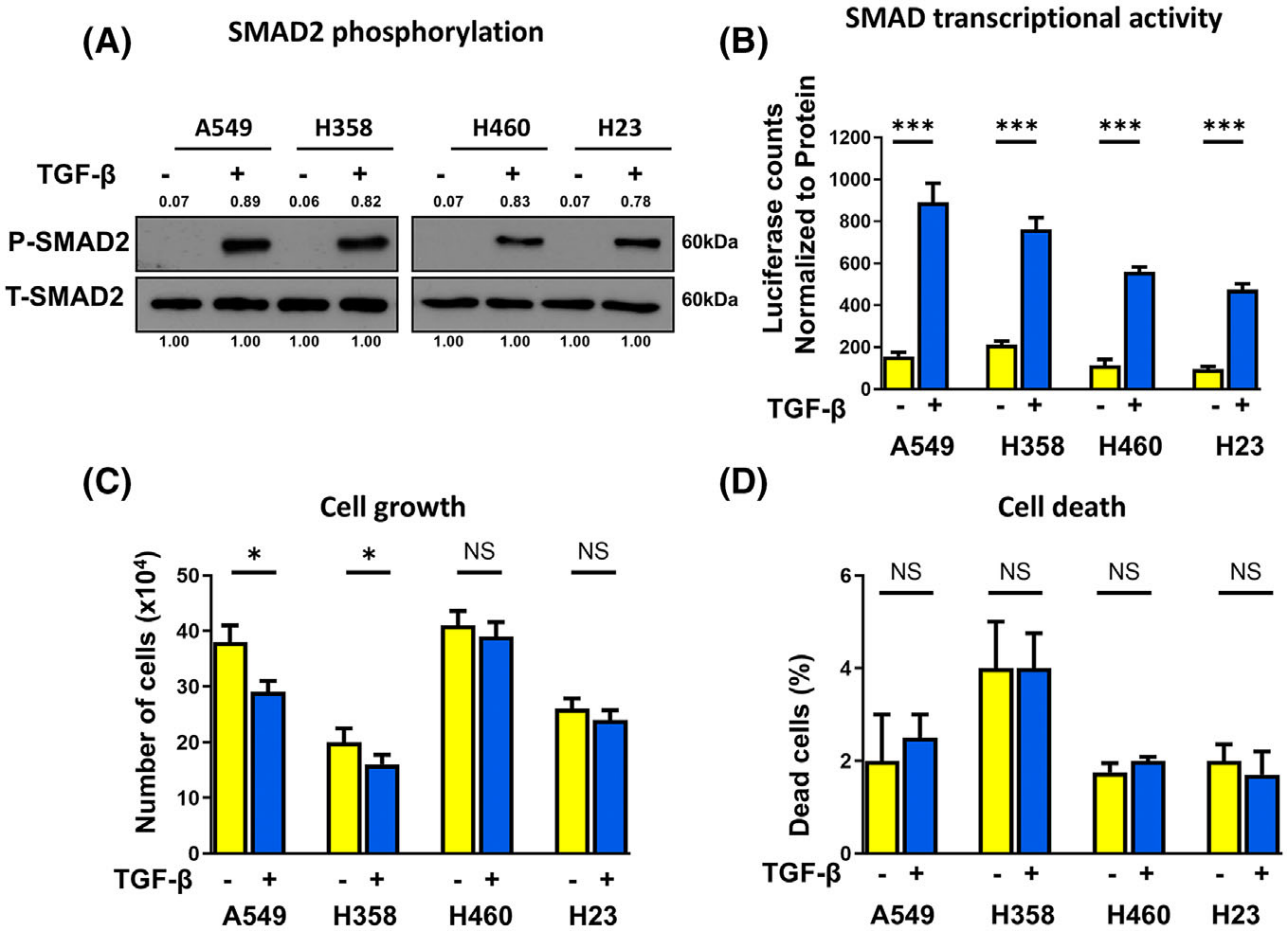
Lung cancer cells are resistant to TGF‐β–induced cell death. (A) Indicated lung cancer cell lines were treated with or without TGF‐β1 (5 ng/mL) for 1 h, and phospho‐SMAD2 and total SMAD2 protein levels were assessed by Western blotting. Band intensities of phospho‐SMAD2 were quantified and normalized to the corresponding total SMAD2 loading control. Normalized protein expression values are indicated above each respective band. (B) Indicated cell lines were transfected with a SMAD‐binding element (SMAD‐BE) luciferase reporter construct and assessed for luciferase activity after 4 h of treatment with or without TGF‐β1 (5 ng/mL). (C) Indicated cell lines were treated with or without TGF‐β1 (5 ng/mL) and analyzed for cell proliferation by cell counting. (D) Indicated cell lines were treated with or without TGF‐β1 (5 ng/mL) and analyzed for cell death by trypan blue exclusion assay. Statistical analysis was performed using a two‐tailed unpaired Welch's *t*‐test. Bars represent mean ± SD from *n* = 3 biological replicates. Statistical significance was defined as NS, not significant; *P < 0.05; ***P < 0.001.

TGF‐β typically functions as a tumor suppressor by inhibiting proliferation and inducing apoptosis in epithelial cells. We examined TGF‐β signaling and its effects on cell growth and survival in four lung cancer cell lines (A549, H358, H460, H23). All the cell lines displayed intact TGF‐β signaling, as indicated by SMAD2 phosphorylation and SMAD‐responsive transcriptional activity at 1‐ and 4‐h poststimulation, respectively (Fig. 1A,B). Treatment with TGF‐β1 up to 96 h moderately reduced cell proliferation, as assessed by counting cell numbers using a Coulter counter, in A549 and H358 cells but had no effect on H460 or H23 (Fig. 1C). Importantly, none of the cell lines exhibited cell death in response to TGF‐β, as determined by trypan blue exclusion (Fig. 1D), suggesting a broad resistance in lung cancer cell lines.

### 3.2 E2A silencing restores TGF‐β‐induced apoptosis but does not affect EMT in mutant KRAS driven cells

**Fig. 2:**
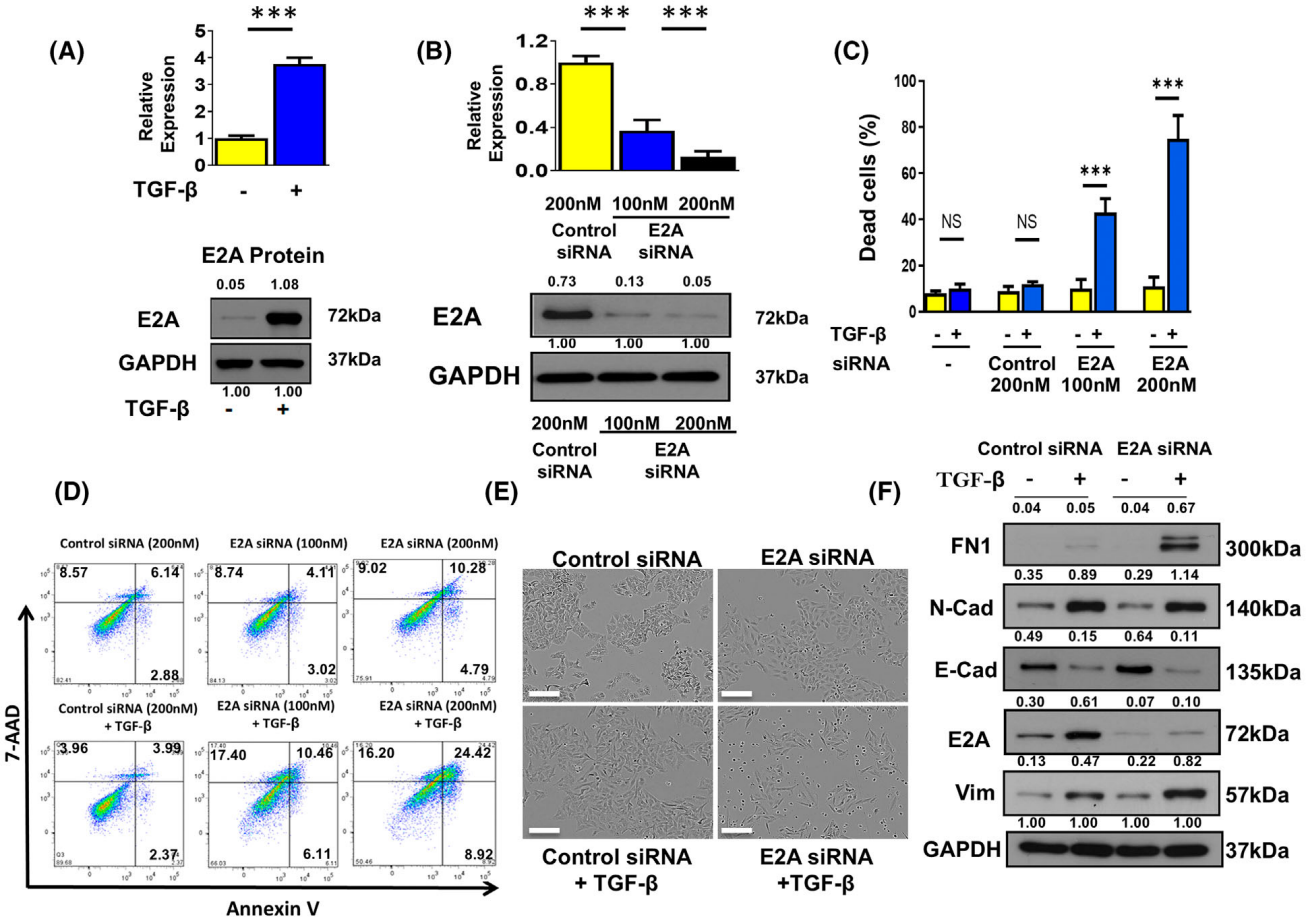
siRNA‐mediated inhibition of E2A restores TGF‐β‐induced apoptosis but does not affect TGF‐β‐induced EMT in lung cancer cells. (A) A549 cells were serum‐starved for 24 h and then treated with or without TGF‐β1 (5 ng/mL). E2A mRNA (top) and protein (bottom) expression levels were assessed by qPCR and Western blotting, respectively, after 72 h. GAPDH was used as a loading control. Protein band intensities were quantified and normalized to the corresponding GAPDH band, and the normalized expression values are shown above each band. (B) A549 cells were transfected with 200 nm control siRNA or 100/200 nM E2A siRNA. E2A mRNA (top) and protein (bottom) expression levels were assessed by qPCR and Western blotting, respectively, after 72 h. GAPDH was used as a loading control. Protein band intensities were quantified and normalized to the corresponding GAPDH band, and the normalized expression values are shown above each band. (C) A549 cells were transfected with control or E2A siRNA, stimulated with TGF‐β1, and cell death was assessed by trypan blue exclusion assay. (D) A549 cells were transfected with control or E2A siRNA at the indicated concentrations, treated with or without TGF‐β1 (5 ng/mL) for 72 h, and assessed for apoptosis by Annexin V/7‐AAD staining by flow cytometry. (E) Cell morphology was examined after 72 h of TGF‐β1 treatment using an EVOS bright‐field inverted microscope at 10 × magnification. Scale bars = 60 μm. (F) A549 cells were transfected with 200 nm of control or E2A siRNA, stimulated with TGF‐β1 (5 ng/mL) for 72 h. Western blot analysis of EMT markers: Fibronectin (FN1), N‐cadherin, E‐cadherin, and vimentin, E2A, and GAPDH. Protein band intensities were quantified and normalized to the corresponding GAPDH band, and the normalized expression values are shown above each band. Statistical analysis was performed using a two‐tailed unpaired Welch's *t*‐test. Bars represent mean ± SD, with *n* = 3 biological replicates. Statistical significance was defined as *NS*, not significant; ***P < 0.001.

Stimulation with TGF‐β1 significantly upregulated E2A mRNA and protein levels in A549 cells (Fig. 2A). Knockdown of E2A via siRNA effectively reduced E2A expression (Fig. 2B), and significantly sensitized A549 cells to TGF‐β‐induced cell death as assessed by Trypan blue exclusion assay (Fig. 2C). The observed cell death is due to induction of apoptosis as indicated by the increase in the proportion of Annexin V/7‐AAD‐positive cells by threefold compared to control siRNA as assessed by flow cytometry (Fig. 2D). Notably, E2A knockdown did not interfere with TGF‐β‐induced epithelial‐to‐mesenchymal transition (EMT). A549 cells maintained their EMT phenotype following TGF‐β1 treatment, as assessed by cell morphology (Fig. 2E) and expression of epithelial and mesenchymal markers (Fig. 2F). These findings indicate that E2A selectively mediates resistance to TGF‐β‐induced apoptosis without affecting EMT.

**Fig. 3:**
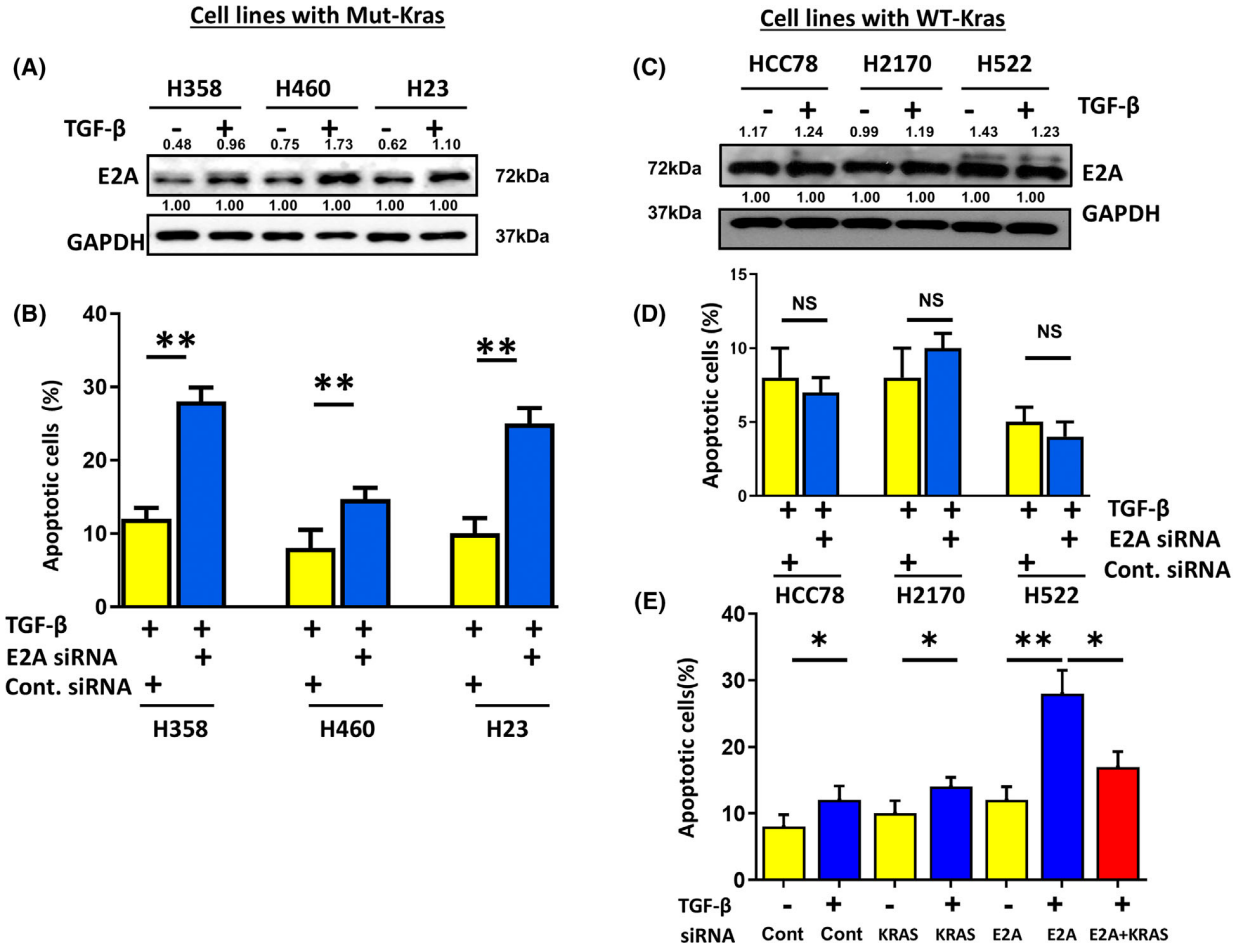
E2A silencing restores TGF‐β–induced apoptosis in cells with mutant KRAS but not wild‐type KRAS. (A) Indicated cell lines with mutant KRAS (mut‐KRAS) were treated with or without TGF‐β1 (5 ng/mL) for 72 h. Protein lysates were analyzed by Western blotting for E2A and GAPDH. Band intensities were quantified and normalized to the corresponding GAPDH loading control. The normalized protein expression values are shown above each respective band. (B) Indicated cell lines with mutant KRAS (mut‐KRAS) transfected with 200 nm of control or E2A siRNA were stimulated with TGF‐β1 (5 ng/mL) for 72 h. Apoptosis was assessed by Annexin V/7‐AAD staining. (C) Indicated cell lines with wild‐type KRAS (WT‐KRAS) were treated and analyzed as in (A). (D) Indicated cell lines with wild‐type KRAS (WT‐KRAS) transfected with 200 nm of control or E2A siRNA were stimulated with TGF‐β1 (5 ng/mL) for 72 h. Apoptosis was assessed by Annexin V/7‐AAD staining. (E) A549 cells were transfected with 200 nm of control, KRAS, or E2A siRNA and stimulated with or without TGF‐β1 (5 ng/mL). Apoptosis was assessed after 72 h by Annexin V/7‐AAD staining. Statistical analysis was performed using a two‐tailed unpaired Welch's *t*‐test. Bars represent mean ± SD, with *n* = 3 biological replicates. Statistical significance was defined as *NS*, not significant; *P < 0.05; **P < 0.01.

The TGF‐β induced E2A expression (Fig. 3A) and restoration of TGF‐β‐induced apoptosis with E2A silencing (Fig. S1A) were also observed in H358, H460, and H23 NSCLC cell lines (Fig. 3B), which are all mutant KRAS driven, such as A549 cells. Interestingly, cell lines that harbor wild‐type KRAS (HCC78, H2170, H522), though express E2A, did not upregulate its expression in response to TGF‐β (Fig. 3C). Like mutant KRAS‐driven cell lines, wild‐type KRAS lines also had intact TGF‐β signaling as assessed by SMAD2/3 transcriptional activity (Fig. S1B), and SMAD2 phosphorylation (Fig. S1C). However, E2A silencing had no effect on TGF‐β‐induced apoptosis (Fig. 3D). Interestingly, there is no difference in the base levels of total‐SMAD3, phosphorylated‐SMAD3 (Fig. S1D) as well as E2A expression (Fig. S1E) between mutant KRAS (H358) and wild‐type KRAS (H522) lines. siRNA mediated inhibition of KRAS in A549 cells significantly reversed the effect of E2A silencing on TGF‐β‐induced apoptosis (Fig. 3E), demonstrating the mutant KRAS dependency.

### 3.3 TGF‐β1 induces E2A expression in a SMAD3‐dependent manner

We next investigated the mechanism of TGF‐β‐induced E2A expression. Upon TGF‐β1 treatment (5 ng/mL, 72 h), E2A expression increased at both mRNA and protein levels in all mutant KRAS lung cancer cell lines tested (Figs. 2A and 3A). To examine SMAD dependency, A549 cells were transfected with siRNAs targeting SMAD2 or SMAD3 and stimulated with TGF‐β. Knockdown of SMAD3, but not SMAD2, abrogated TGF‐β‐induced E2A mRNA (Fig. S2A) or protein (Fig. S2B) expression, indicating a SMAD3‐dependent mechanism. Previously validated siRNAs achieved 80–90% knockdown efficiency [16].

### 3.4 E2A inhibition increases mitochondrial outer membrane permeability (MOMP) and caspase 3 activation in response to TGF‐β1 treatment

**Fig. 4:**
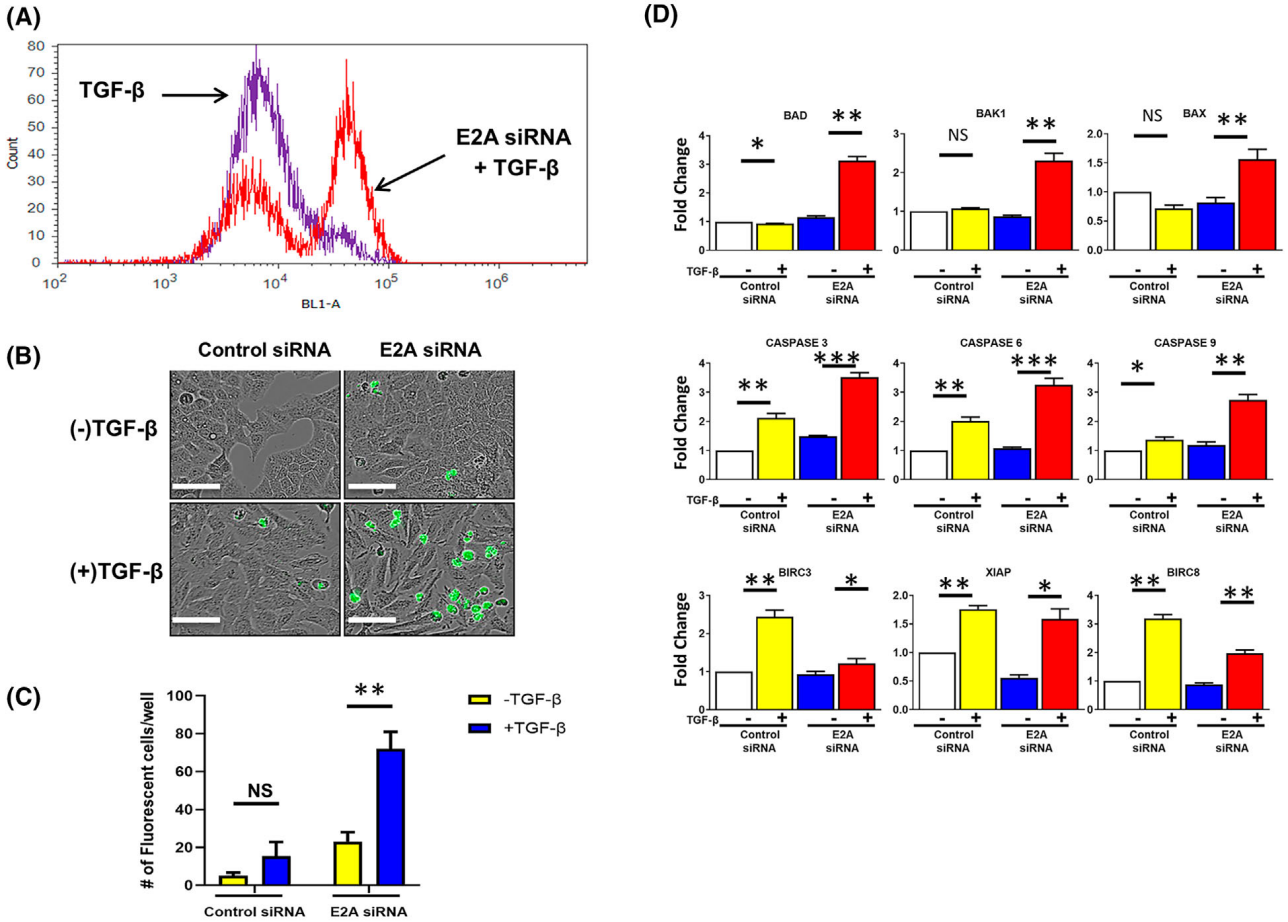
E2A knockdown impairs mitochondrial outer membrane permeability (MOMP), increases caspase‐3 activity, and alters expression of apoptosis regulators in response to TGF‐β. (A) A549 cells transfected with control or E2A siRNA were treated with or without TGF‐β1 (5 ng/mL) for 72 h. MOMP was evaluated using MitoProbe JC‐1 dye and flow cytometry. (B) A549 cells transfected with control or E2A siRNA were treated with or without TGF‐β1 (5 ng/mL) for 72 h. Caspase‐3 activation was monitored using NucView 488 fluorescent substrate (green) and live‐cell imaging. Scale bars = 60 μm. Time‐lapse videos are shown in the supplementary video files. (C) Caspase‐3–positive cells shown in (B) were quantified using IncuCyte analysis software. (D) Gene expression of apoptosis‐related genes was assessed using a pathway‐specific qPCR following E2A knockdown and TGF‐β1 treatment. Statistical analysis was performed using a two‐tailed unpaired Welch's *t*‐test. Bars represent mean ± SD, with *n* = 3 biological replicates. Statistical significance was defined as *NS*, not significant; *P < 0.05; **P < 0.01; ***P < 0.001.

Loss of mitochondrial inner membrane potential, leading to increased mitochondrial outer membrane permeability (MOMP), is a hallmark of apoptosis. In A549 cells, E2A knockdown in the presence of TGF‐β1 caused a significant increase in MOMP (Fig. 4A), as measured by shifts in JC‐1 fluorescence by flow cytometry. Caspase‐3 activation was evaluated using the nucview DEVD‐based fluorescent substrate, and E2A knockdown substantially elevated caspase‐3 activity in TGF‐β1–treated cells (Fig. 4B, Fig. 4C, and supplementary videos).

Consistently, pathway‐specific apoptosis SuperArray analysis, followed by qPCR validation of select genes, showed that E2A knockdown in the presence of TGF‐β1 increased expression of pro‐apoptotic genes (Bad, Bak1, Bax) and executioner caspases (Casp3, Casp6, Casp9), while reducing the expression of anti‐apoptotic factors BIRC3, BIRC8, and XIAP (Fig. 4D and Table S1).

Together, these findings suggest that E2A regulates the apoptotic threshold by modulating the balance of pro‐ and anti‐apoptotic gene expression during TGF‐β1–induced apoptosis.

### 3.5 E2A inhibition increases radiation‐induced growth suppression through TGF‐β signaling

**Fig. 5:**
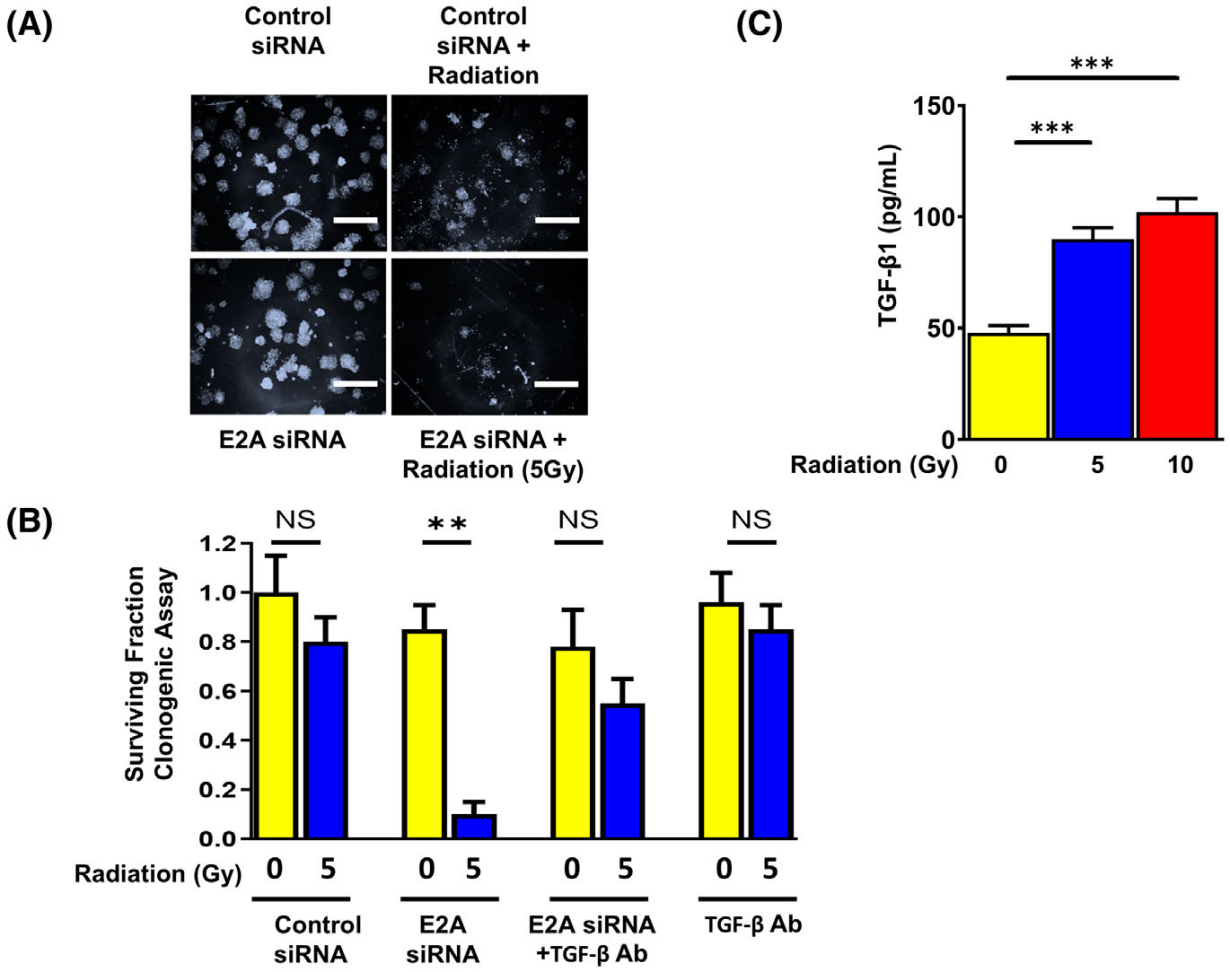
E2A knockdown enhances radiation‐induced growth inhibition of lung cancer cells through increased secretion of TGF‐β. (A, B) A549 cells were transfected with control or E2A siRNA and irradiated with 5 Gy. After 24 h, surviving cells were replated and allowed to form colonies for 7 days. (A) Colonies were visualized using an EVOS bright‐field inverted microscope at 2 × magnification. Scale bars = 100 μm. (B) Surviving fraction was quantified by manual colony counting (C) A549 cells were irradiated with 0, 5, or 10 Gy, and TGF‐β levels in culture supernatants were measured after 48 h by ELISA. Statistical analysis was performed using a two‐tailed unpaired Welch's *t*‐test. Bars represent mean ± SD, with *n* = 3 biological replicates. Statistical significance was defined as *NS*, not significant; **P < 0.01; ***P < 0.001.

To determine the therapeutic relevance of E2A targeting, we examined its impact on radiation‐induced growth inhibition. A549 cells transfected with control or E2A siRNA were exposed to 5 Gy irradiation, and colony formation was assessed by clonogenic assay. Radiation alone produced only a modest reduction in colony growth, whereas E2A knockdown caused a dramatic ~ 90% decrease (Fig. 5A,B). As radiation is known to stimulate TGF‐β expression, we confirmed that irradiated A549 cells secreted increasing levels of TGF‐β1 in a dose‐dependent manner as measured by ELISA of conditioned media (Fig. 5C). Importantly TGF‐β neutralizing antibody blocked E2A siRNA‐mediated inhibition of colony formation (Fig. 5B). These findings support the clinical potential of targeting E2A to restore TGF‐β‐induced apoptosis and enhance therapeutic efficacy of radiation.

### 3.6 E2A is differentially expressed in lung adenocarcinomas with elevated expression in tumors harboring mutant KRAS compared to those with wild‐type KRAS

**Fig. 6:**
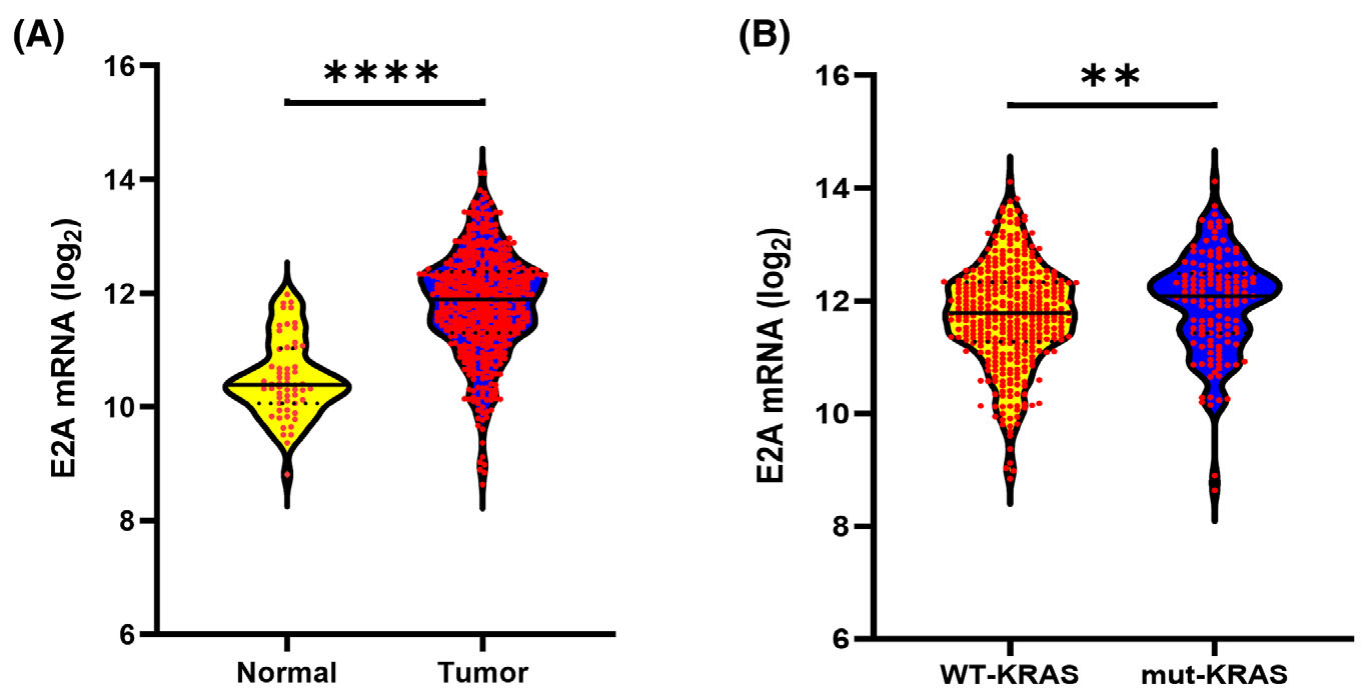
E2A expression in primary tumors versus normal lung tissue and mutant KRAS versus wild‐type KRAS tumors in patients with lung adenocarcinoma. (A) E2A gene expression in lung adenocarcinoma tumors vs. adjacent normal lung tissue from TCGA‐LUAD dataset was analyzed using Mann–Whitney test (tumors *n* = 513, normal *n* = 58). Y‐axis represents log_2_ median‐centered intensity (****P < 0.0001). (B) E2A expression in tumors from the same dataset was analyzed using the Mann–Whitney test with wild‐type KRAS (*n* = 375) vs. mutant KRAS (*n* = 138) (**P < 0.01).

To assess the clinical relevance of E2A, we analyzed its mRNA expression in normal and tumor lung tissues using data from The Cancer Genome Atlas (TCGA). E2A was significantly overexpressed in lung adenocarcinoma (LUAD) tissues compared to normal lung tissues (*n* = 513 tumors vs. 58 normal; *p* < 0.0001). Moreover, E2A expression was significantly higher in tumors harboring mutant KRAS compared to those with wild‐type KRAS (WT: *n* = 375, mut: *n* = 138, *p* = 0.0093) (Fig. 6A,B). These findings are consistent with a tumor‐promoting role for E2A in mutant KRAS driven lung cancer.

## 4 Discussion

TGF‐β remains one of the most paradoxical pathways in cancer biology, acting as a tumor suppressor in early stages and promoting tumor progression in advanced disease [1]. Although canonical SMAD signaling [17] remains intact in most non‐small cell lung cancers (NSCLCs), including KRAS‐mutant subtypes, these cells exhibited marked resistance to TGF‐β‐induced apoptosis. Our findings uncover a mechanism underlying this resistance, which is a TGF‐β–induced upregulation of E2A in a SMAD3‐dependent fashion, to suppress mitochondrial apoptosis without impairing EMT. Notably, this mechanism is specific to mutant KRAS‐harboring NSCLC, thereby identifying E2A as a context‐dependent modulator of tumor cell apoptosis.

Recent work has emphasized the reprogramming of TGF‐β responses by oncogenic KRAS, which may shift TGF‐β signaling from cytostatic and apoptotic to pro‐survival phenotype [18, 19]. Our study supports this paradigm by showing that E2A can serve as an effector of the rewired TGF‐β response in mutant KRAS lung cancer. The fact that E2A knockdown restores apoptosis specifically in mutant KRAS cell lines, and that this effect is abrogated by KRAS silencing, underscores a functional KRAS–TGF‐β–E2A axis critical for cell survival. Furthermore, mutant KRAS has been shown to promote secretion of immunosuppressive cytokines including TGF‐β, suggesting a feedback mechanism whereby KRAS promotes both TGF‐β signaling and E2A induction to avoid apoptotic outcomes and reinforce tumor progression [20].

It is tempting to speculate that these findings may hold direct relevance for the therapeutic landscape of KRAS‐mutant NSCLC, particularly in the context of mutant KRAS inhibitors such as sotorasib and adagrasib. While these agents have demonstrated clinical benefit, objective response rates remain modest, and resistance emerges rapidly [21, 22, 23]. Resistance mechanisms include bypass pathway activation (e.g., MET, EGFR), secondary KRAS mutations, and rewiring of apoptotic machinery [21, 24]. Our data suggest that E2A could represent a novel contributor to apoptotic resistance in mutant KRAS tumors, especially under therapeutic pressure. The upregulation of E2A may shield cells from apoptotic stress induced not only by TGF‐β but also by targeted therapies, potentially contributing to their acquired resistance [21].

The ability of E2A inhibition to sensitize mutant KRAS NSCLC cells to radiation is also noteworthy. Radiation is known to increase TGF‐β production in the tumor microenvironment, which promotes immune evasion, fibrosis, and metastasis in some contexts [25, 26, 27].

By disrupting E2A, we were able to unmask the cytotoxic potential of endogenously induced TGF‐β following radiation exposure. This suggests that combinatorial strategies integrating E2A inhibition with radiation could restore tumor suppression without compromising the pleiotropic benefits of TGF‐β inhibition on the tumor microenvironment and immune function [26, 28].

E2A knockdown had no detectable impact on TGF‐β–induced EMT as evidenced by retained mesenchymal morphology and expression of canonical EMT markers such as vimentin and N‐cadherin. This is surprising given earlier studies identifying E2A as a repressor of E‐cadherin and a promoter of EMT in epithelial cells [15, 29]. Our data suggest that E2A may play dual and context‐dependent roles in cancer biology, promoting EMT in some settings while suppressing apoptosis in others. This dichotomy raises the possibility that E2A is part of a larger network of transcription factors whose functions are modulated by the context of TGF‐β signaling, oncogenic drivers, or cellular lineage involved. This also presents a therapeutic opportunity where E2A inhibition could restore apoptotic sensitivity in KRAS‐mutant tumors while preserving the EMT‐related functions that may be contextually beneficial or targetable via other strategies [30].

Finally, our analysis of clinical datasets reveals that E2A expression is significantly elevated in LUAD compared to normal lung tissues. Consistent with our cell line data, we observed elevated E2A expression in the mutant KRAS compared to wild‐type KRAS tumors. Although the oncogenic fusion proteins of E2A are well‐studied in hematologic malignancies [14, 31], E2A is rarely mutated or translocated in solid tumors. Our data suggest that E2A may be oncogenic in solid tumors through a distinct mechanism involving suppression of TGF‐β‐mediated apoptosis. This regulation may be primarily transcriptional or post‐transcriptional, possibly involving KRAS and/or SMAD3‐dependent chromatin remodeling, both of which warrant further investigation.

## 5 Conclusion

In conclusion, our study defines a novel KRAS–TGF‐β–E2A axis that enables NSCLC cells to evade apoptosis while retaining TGF‐β‐driven EMT and survival signals. By doing so, E2A contributes to the uncoupling of tumor‐suppressive and tumor‐promoting TGF‐β responses, enabling cancer progression in the context of otherwise intact TGF‐β signaling. By targeting E2A, we demonstrate restoration of TGF‐β‐induced mitochondrial apoptosis and radiosensitization in KRAS‐mutant lung cancer cells. These findings support a new therapeutic strategy aimed at reactivating the tumor‐suppressive arm of TGF‐β signaling, particularly in the setting of KRAS‐driven oncogenesis and resistance to targeted therapies. Future efforts should explore E2A as both a biomarker and therapeutic vulnerability in rational combinations with KRAS inhibitors, radiation, and possibly immunomodulatory agents.

## Conflict of interest

The authors declare no conflict of interest.

## Author contributions

SC and VK designed the experiments; VK and SC wrote the manuscript. SC, SK, and ZW performed the experiments, analyzed the data, and prepared the figures. VK conceived the project, secured funding, supervised the study, and edited the manuscript. All authors reviewed and approved the final version.

## Declaration

Large language model (ChatGPT) was used to improve language and readability; final content was reviewed and approved by all the authors.

## Supporting information


**Table S1.** qPCR SuperArray analysis of apoptosis‐related gene expression.

**Fig. S1.** Validation of siRNA‐mediated knockdown of E2A in mutant KRAS cell lines and SMAD2 activation in wild‐type KRAS cell lines.

**Fig. S2.** TGF‐β1 induces E2A expression in a SMAD3‐dependent manner.

**Video S1.** Supplementary video files: Time‐lapse videos of caspase‐3 activation in A549 cells treated with E2A or control siRNA with or without TGF‐β1 treatment.

**Video S2.** 2‐Control‐siRNA‐TGF.

**Video S3.** 3‐E2A‐siRNA.

**Video S4.** 4‐E2A‐siRNA‐TGF.

**Data S1.** Supplementary Legends.

## Data Availability

All data generated or analyzed during this study are included in this article or provided in supplementary information. Additional data are available from the corresponding author upon reasonable request.
