# Supplementary material for: E2A selectively regulates TGF‐β–induced apoptosis in KRAS‐mutant non‐small cell lung cancer
Source: Mol Oncol. 2026 Mar 17;20(7):1877–88. doi: 10.1002/1878-0261.70236 (PMC13352951; doi:10.1002/1878-0261.70236)
Supplement: Supplementary file 1 — Table S1. qPCR SuperArray analysis of apoptosis‐related gene expression. [file MOL2-20-1877-s008.pdf]

Gene Name	Samples				Delta Ct				2 Delta Ct				Fold Change				Normalized	Normalized
Gene Symbol	Sample 1	Sample 2	Sample 3	Sample 4	Control	Control+TGF	E2A	E2A+TGF	F2-F2	G2-F2	H2-F2	I2-F2	Control	Control+TGF	E2A	E2A+TGF	E2A/Control	E2A+TGF/Control+TGF
ABL1	29.66	30.10	29.67	29.18	8.27	7.07	8.26	6.65	0.00	-1.21	-0.02	-1.62	1.00	2.31	***1.01***	3.07	1.01	1.33
AKT1	30.46	32.13	30.74	31.18	9.07	9.09	9.33	8.65	0.00	0.02	0.26	-0.42	1.00	0.99	***0.84***	1.34	0.84	1.36
APAF1	31.46	32.57	31.40	31.58	10.07	9.53	9.99	9.05	0.00	-0.54	-0.08	-1.02	1.00	1.45	***1.06***	2.02	1.06	1.40
BAD	29.98	31.74	29.87	29.54	8.59	8.71	8.46	7.01	0.00	0.12	-0.13	-1.58	1.00	0.92	***1.10***	2.98	1.10	3.24
BAG1	28.59	30.66	28.84	30.10	7.20	7.62	7.43	7.58	0.00	0.42	0.22	0.37	1.00	0.75	***0.86***	0.77	0.86	1.03
BAG3	27.83	32.02	27.35	27.88	6.44	8.98	5.94	5.35	0.00	2.54	-0.50	-1.09	1.00	0.17	***1.42***	2.13	1.42	12.38
BAG4	30.11	32.05	30.21	30.84	8.72	9.01	8.80	8.31	0.00	0.29	0.08	-0.41	1.00	0.82	***0.95***	1.33	0.95	1.62
BAK1	29.69	31.25	29.91	29.74	8.31	8.22	8.50	7.22	0.00	-0.09	0.19	-1.09	1.00	1.06	***0.88***	2.13	0.88	2.01
BAX	29.99	32.00	30.31	30.43	8.60	8.96	8.90	7.91	0.00	0.36	0.29	-0.70	1.00	0.78	***0.82***	1.62	0.82	2.07
BCL10	28.81	29.51	29.24	28.53	7.43	6.48	7.83	6.01	0.00	-0.95	0.40	-1.42	1.00	1.93	***0.76***	2.68	0.76	1.38
BCL2	31.70	33.36	32.42	32.23	10.31	10.32	11.01	9.70	0.00	0.01	0.70	-0.61	1.00	0.99	***0.62***	1.52	0.62	1.54
BCL2A1	30.10	29.89	30.08	26.58	8.72	6.85	8.67	4.06	0.00	-1.86	-0.04	-4.66	1.00	3.64	***1.03***	25.28	1.03	6.95
BCL2L1	30.68	32.54	30.67	31.12	9.30	9.51	9.26	8.60	0.00	0.21	-0.04	-0.70	1.00	0.87	***1.03***	1.62	1.03	1.88
BCL2L10	30.67	30.63	30.85	31.06	9.29	7.59	9.44	8.54	0.00	-1.70	0.15	-0.75	1.00	3.24	***0.90***	1.69	0.90	0.52
BCL2L11	28.67	29.30	28.45	28.99	7.28	6.26	7.04	6.47	0.00	-1.02	-0.24	-0.81	1.00	2.03	***1.18***	1.76	1.18	0.87
BCL2L2	29.31	30.58	29.42	29.32	7.92	7.55	8.01	6.79	0.00	-0.38	0.09	-1.13	1.00	1.30	***0.94***	2.19	0.94	1.69
BCLAF1	27.89	28.69	27.44	27.69	6.51	5.65	6.03	5.17	0.00	-0.85	-0.48	-1.34	1.00	1.81	***1.39***	2.53	1.39	1.40
BFAR	29.75	30.06	29.20	29.28	8.36	7.02	7.79	6.75	0.00	-1.34	-0.58	-1.61	1.00	2.53	***1.49***	3.06	1.49	1.21
BID	28.68	29.86	28.75	28.78	7.30	6.82	7.34	6.26	0.00	-0.48	0.04	-1.04	1.00	1.39	***0.98***	2.05	0.98	1.48
BIK	35.60	37.23	36.08	35.92	14.22	14.19	14.67	13.40	0.00	-0.02	0.45	-0.82	1.00	1.02	***0.73***	1.76	0.73	1.74
NAIP	29.59	29.84	29.33	29.51	8.20	6.81	7.92	6.99	0.00	-1.39	-0.29	-1.21	1.00	2.63	***1.22***	2.32	1.22	0.88
BIRC2	29.66	31.12	29.86	28.48	8.27	8.08	8.45	5.95	0.00	-0.19	0.18	-2.32	1.00	1.14	***0.88***	4.99	0.88	4.39
BIRC3	31.14	31.44	31.15	31.85	9.76	8.40	9.74	9.33	0.00	-1.35	-0.02	-0.43	1.00	2.56	***1.02***	1.35	1.02	0.53
XIAP	31.49	32.28	32.55	32.11	10.10	9.24	11.14	9.59	0.00	-0.86	1.04	-0.52	1.00	1.82	***0.49***	1.43	0.49	0.79
BIRC6	30.49	30.45	29.99	29.17	9.10	7.42	8.58	6.65	0.00	-1.69	-0.52	-2.45	1.00	3.22	***1.43***	5.47	1.43	1.70
BIRC8	30.58	30.52	30.78	30.77	9.20	7.48	9.37	8.25	0.00	-1.71	0.17	-0.95	1.00	3.28	***0.89***	1.93	0.89	0.59
BNIP1	30.66	32.58	30.61	32.23	9.28	9.55	9.20	9.70	0.00	0.27	-0.07	0.43	1.00	0.83	***1.05***	0.74	1.05	0.90
BNIP2	29.26	30.07	29.44	30.88	7.87	7.03	8.03	8.36	0.00	-0.84	0.15	0.49	1.00	1.79	***0.90***	0.71	0.90	0.40
BNIP3	26.01	27.90	25.62	26.81	4.62	4.86	4.21	4.29	0.00	0.24	-0.41	-0.33	1.00	0.85	***1.33***	1.26	1.33	1.49
BNIP3L	27.95	29.48	28.24	28.61	6.56	6.45	6.83	6.09	0.00	-0.11	0.26	-0.47	1.00	1.08	***0.83***	1.39	0.83	1.28
BRAF	30.66	30.96	30.63	30.55	9.28	7.93	9.22	8.02	0.00	-1.35	-0.06	-1.26	1.00	2.55	***1.04***	2.39	1.04	0.94
NOD1	29.72	30.54	30.42	30.19	8.34	7.51	9.01	7.66	0.00	-0.83	0.68	-0.68	1.00	1.78	***0.63***	1.60	0.63	0.90
CARD6	Indetermine	Indetermine	39.83	38.83	#VALUE!	#VALUE!	18.42	16.30	#VALUE!	#VALUE!	#VALUE!	#VALUE!	#VALUE!	#VALUE!	***#VALUE!***	#VALUE!	#VALUE!	#VALUE!
CARD8	32.54	31.76	31.49	31.81	11.15	8.73	10.08	9.29	0.00	-2.42	-1.07	-1.87	1.00	5.37	***2.10***	3.65	2.10	0.68
CASP1	31.22	31.60	31.48	31.71	9.83	8.57	10.07	9.19	0.00	-1.27	0.23	-0.65	1.00	2.41	***0.85***	1.57	0.85	0.65
CASP10	30.85	31.39	30.84	30.76	9.47	8.35	9.43	8.24	0.00	-1.11	-0.04	-1.23	1.00	2.16	***1.03***	2.35	1.03	1.08
CASP14	31.30	32.02	31.38	31.63	9.91	8.99	9.97	9.11	0.00	-0.92	0.06	-0.81	1.00	1.89	***0.96***	1.75	0.96	0.92
CASP2	31.05	31.18	31.25	31.32	9.66	8.14	9.84	8.80	0.00	-1.52	0.18	-0.86	1.00	2.86	***0.88***	1.82	0.88	0.64
CASP3	30.20	30.88	29.68	29.59	8.81	7.84	8.27	7.07	0.00	-0.97	-0.55	-1.74	1.00	1.96	***1.46***	3.35	1.46	1.71
CASP4	29.27	30.56	28.67	29.22	7.88	7.52	7.26	6.70	0.00	-0.36	-0.63	-1.19	1.00	1.28	***1.54***	2.28	1.54	1.77
CASP5	31.81	32.56	32.12	32.03	10.42	9.53	10.71	9.51	0.00	-0.90	0.29	-0.92	1.00	1.86	***0.82***	1.89	0.82	1.01
CASP6	30.30	30.97	30.17	29.84	8.91	7.94	8.76	7.32	0.00	-0.97	-0.15	-1.59	1.00	1.96	***1.11***	3.01	1.11	1.53
CASP7	30.13	30.72	30.19	30.26	8.75	7.69	8.78	7.74	0.00	-1.06	0.04	-1.01	1.00	2.09	***0.98***	2.01	0.98	0.96
CASP8	29.62	30.30	29.35	29.41	8.24	7.27	7.94	6.89	0.00	-0.97	-0.30	-1.35	1.00	1.96	***1.23***	2.55	1.23	1.30
CASP9	29.26	30.50	29.10	29.04	7.87	7.46	7.69	6.52	0.00	-0.41	-0.18	-1.36	1.00	1.33	***1.14***	2.56	1.14	1.92
CD40	30.39	31.44	30.60	30.79	9.01	8.41	9.19	8.27	0.00	-0.60	0.19	-0.74	1.00	1.51	***0.88***	1.67	0.88	1.10
CD40LG	31.75	32.26	31.74	32.14	10.37	9.23	10.33	9.62	0.00	-1.14	-0.04	-0.75	1.00	2.21	***1.03***	1.69	1.03	0.76
CFLAR	30.33	30.35	29.69	29.16	8.94	7.32	8.28	6.64	0.00	-1.62	-0.66	-2.30	1.00	3.08	***1.58***	4.93	1.58	1.60
CIDEA	30.55	31.93	31.48	31.64	9.16	8.89	10.07	9.12	0.00	-0.27	0.91	-0.04	1.00	1.21	***0.53***	1.03	0.53	0.86
CIDEB	31.28	32.68	31.22	31.89	9.89	9.65	9.81	9.37	0.00	-0.24	-0.08	-0.52	1.00	1.18	***1.06***	1.44	1.06	1.21
CRADD	30.28	30.22	29.35	29.34	8.90	7.19	7.94	6.82	0.00	-1.71	-0.96	-2.08	1.00	3.26	***1.94***	4.22	1.94	1.29
DAPK1	26.94	31.15	27.18	29.29	5.56	8.11	5.77	6.76	0.00	2.56	0.22	1.21	1.00	0.17	***0.86***	0.43	0.86	2.55
DFFA	30.36	31.44	30.26	30.31	8.97	8.41	8.85	7.79	0.00	-0.56	-0.12	-1.19	1.00	1.48	***1.09***	2.28	1.09	1.54
FADD	30.31	31.12	32.10	30.51	8.92	8.08	10.69	7.98	0.00	-0.84	1.76	-0.94	1.00	1.79	***0.29***	1.92	0.29	1.07
FAS	31.36	31.84	30.78	30.72	9.98	8.81	9.37	8.20	0.00	-1.17	-0.61	-1.78	1.00	2.25	***1.52***	3.43	1.52	1.53
FASLG	31.12	31.93	31.11	31.35	9.74	8.90	9.70	8.82	0.00	-0.84	-0.04	-0.92	1.00	1.79	***1.03***	1.89	1.03	1.06
GADD45A	31.00	31.69	30.87	29.82	9.62	8.66	9.46	7.30	0.00	-0.96	-0.16	-2.32	1.00	1.95	***1.12***	5.00	1.12	2.57
HRK	29.84	30.82	29.82	30.05	8.45	7.79	8.41	7.52	0.00	-0.67	-0.05	-0.93	1.00	1.59	***1.03***	1.90	1.03	1.20
IGF1R	25.68	26.17	25.30	25.49	4.30	3.13	3.89	2.97	0.00	-1.17	-0.40	-1.33	1.00	2.24	***1.32***	2.51	1.32	1.12
LTA	31.53	32.58	31.53	31.49	10.14	9.55	10.12	8.96	0.00	-0.60	-0.02	-1.18	1.00	1.51	***1.01***	2.26	1.01	1.50
LTBR	28.75	30.03	27.64	28.85	7.37	6.99	6.23	6.33	0.00	-0.38	-1.14	-1.04	1.00	1.30	***2.20***	2.05	2.20	1.58
MCL1	25.63	25.60	25.55	23.70	4.24	2.56	4.14	1.17	0.00	-1.68	-0.10	-3.07	1.00	3.21	***1.07***	8.41	1.07	2.62
NOL3	28.39	28.72	28.07	27.59	7.00	5.69	6.66	5.06	0.00	-1.31	-0.34	-1.94	1.00	2.48	***1.26***	3.83	1.26	1.54
PYCARD	27.97	28.65	27.94	27.91	6.59	5.61	6.53	5.38	0.00	-0.98	-0.06	-1.20	1.00	1.97	***1.05***	2.30	1.05	1.17
RIPK2	28.33	28.70	28.99	28.12	6.94	5.66	7.58	5.60	0.00	-1.28	0.63	-1.35	1.00	2.43	***0.64***	2.55	0.64	1.05
TNF	31.45	32.52	32.50	31.43	10.06	9.48	11.09	8.90	0.00	-0.58	1.03	-1.16	1.00	1.49	***0.49***	2.23	0.49	1.49
TNFRSF10A	31.47	31.94	31.39	31.75	10.09	8.91	9.98	9.23	0.00	-1.18	-0.11	-0.86	1.00	2.27	***1.08***	1.81	1.08	0.80
TNFRSF10B	29.93	30.91	30.04	29.97	8.55	7.87	8.63	7.45	0.00	-0.68	0.08	-1.10	1.00	1.60	***0.95***	2.15	0.95	1.34
TNFRSF11B	30.57	29.63	32.15	27.19	9.18	6.60	10.74	4.66	0.00	-2.58	1.56	-4.52	1.00	5.99	***0.34***	22.87	0.34	3.82
TNFRSF1A	26.83	27.94	26.43	27.71	5.45	4.91	5.02	5.19	0.00	-0.54	-0.43	-0.26	1.00	1.46	***1.35***	1.20	1.35	0.82
TNFRSF21	30.74	31.71	29.98	31.19	9.35	8.67	8.57	8.67	0.00	-0.68	-0.78	-0.68	1.00	1.60	***1.71***	1.61	1.71	1.00
TNFRSF25	30.93	33.23	31.56	32.18	9.55	10.20	10.15	9.66	0.00	0.65	0.60	0.11	1.00	0.64	***0.66***	0.93	0.66	1.45
CD27	30.57	31.56	31.22	31.09	9.18	8.53	9.81	8.56	0.00	-0.66	0.63	-0.62	1.00	1.58	***0.65***	1.54	0.65	0.98
TNFRSF9	31.90	31.86	31.90	31.72	10.52	8.83	10.49	9.20	0.00	-1.69	-0.03	-1.32	1.00	3.24	***1.02***	2.50	1.02	0.77
TNFSF10	31.28	31.39	30.28	31.59	9.89	8.36	8.87	9.06	0.00	-1.53	-1.02	-0.83	1.00	2.89	***2.03***	1.78	2.03	0.61
CD70	31.36	32.22	31.92	31.03	9.98	9.18	10.51	8.50	0.00	-0.80	0.53	-1.47	1.00	1.74	***0.69***	2.78	0.69	1.60
TNFSF8	30.32	30.93	30.48	30.69	8.93	7.90	9.07	8.16	0.00	-1.03	0.14	-0.77	1.00	2.05	***0.91***	1.71	0.91	0.83
TP53	28.35	29.28	28.45	28.49	6.96	6.25	7.04	5.97	0.00	-0.72	0.07	-1.00	1.00	1.64	***0.95***	1.99	0.95	1.21
TP53BP2	29.80	30.60	29.49	29.19	8.42	7.56	8.08	6.66	0.00	-0.86	-0.34	-1.76	1.00	1.81	***1.27***	3.38	1.27	1.87
TP73	31.15	33.01	31.53	31.74	9.77	9.97	10.12	9.22	0.00	0.21	0.35	-0.55	1.00	0.87	***0.78***	1.46	0.78	1.69
TRADD	29.52	30.73	29.51	29.75	8.14	7.70	8.10	7.23	0.00	-0.44	-0.04	-0.91	1.00	1.36	***1.03***	1.88	1.03	1.38
TRAF2	30.91	32.70	31.39	31.32	9.53	9.66	9.98	8.80	0.00	0.13	0.45	-0.73	1.00	0.91	***0.73***	1.66	0.73	1.82
TRAF3	30.91	31.01	30.94	30.92	9.52	7.97	9.53	8.40	0.00	-1.55	0.01	-1.12	1.00	2.93	***0.99***	2.18	0.99	0.74
TRAF4	32.34	33.34	32.09	32.35	10.96	10.30	10.68	9.83	0.00	-0.65	-0.27	-1.13	1.00	1.57	***1.21***	2.18	1.21	1.39
B2M	25.36	25.64	24.75	24.66	3.98	2.60	3.34	2.14	0.00	-1.37	-0.63	-1.84	1.00	2.59	***1.55***	3.58	1.55	1.38
HPRT1	29.82	30.44	29.56	29.78	8.44	7.40	8.15	7.26	0.00	-1.04	-0.29	-1.18	1.00	2.05	***1.22***	2.27	1.22	1.11
RPL13A	23.37	24.27	23.64	23.99	1.99	1.24	2.23	1.47	0.00	-0.75	0.24	-0.52	1.00	1.68	***0.85***	1.44	0.85	0.85
GAPDH	21.39	23.03	21.41	22.52	0.00	0.00	0.00	0.00	0.00	0.00	0.00	0.00	1.00	1.00	***1.00***	1.00	1.00	1.00

ACTB	23.45	23.14	23.05	22.14	2.06	0.11	1.64	-0.39	0.00	-1.96	-0.42	-2.45	1.00	3.88	***1.34***	5.46	1.34	1.41
HGDC	31.95	32.98	32.47	32.68	10.56	9.95	11.06	10.16	0.00	-0.61	0.50	-0.40	1.00	1.53	***0.71***	1.32	0.71	0.86
RTC	38.30	35.97	38.07	37.82	16.91	12.93	16.66	15.29	0.00	-3.98	-0.25	-1.62	1.00	15.78	***1.19***	3.07	1.19	0.19
RTC	Indetermine	Indetermine	37.24	Indetermine	#VALUE!	#VALUE!	15.83	#VALUE!	#VALUE!	#VALUE!	#VALUE!	#VALUE!	#VALUE!	#VALUE!	***#VALUE!***	#VALUE!	#VALUE!	#VALUE!
RTC	36.59	Indetermine	37.59	37.78	15.20	#VALUE!	16.18	15.25	0.00	#VALUE!	0.98	0.05	1.00	#VALUE!	***0.51***	0.97	0.51	#VALUE!
PPC	22.20	22.25	21.83	21.99	0.81	-0.78	0.42	-0.53	0.00	-1.59	-0.39	-1.34	1.00	3.02	***1.31***	2.54	1.31	0.84
PPC	22.67	22.38	21.93	21.90	1.28	-0.66	0.52	-0.62	0.00	-1.94	-0.77	-1.90	1.00	3.84	***1.70***	3.74	1.70	0.97
PPC	22.92	22.40	22.11	22.09	1.53	-0.63	0.70	-0.43	0.00	-2.17	-0.83	-1.96	1.00	4.49	***1.78***	3.90	1.78	0.87
