# Supplementary figures and images for: E2A selectively regulates TGF‐β–induced apoptosis in KRAS‐mutant non‐small cell lung cancer
Source: Mol Oncol. 2026 Mar 17;20(7):1877–88. doi: 10.1002/1878-0261.70236 (PMC13352951; doi:10.1002/1878-0261.70236)

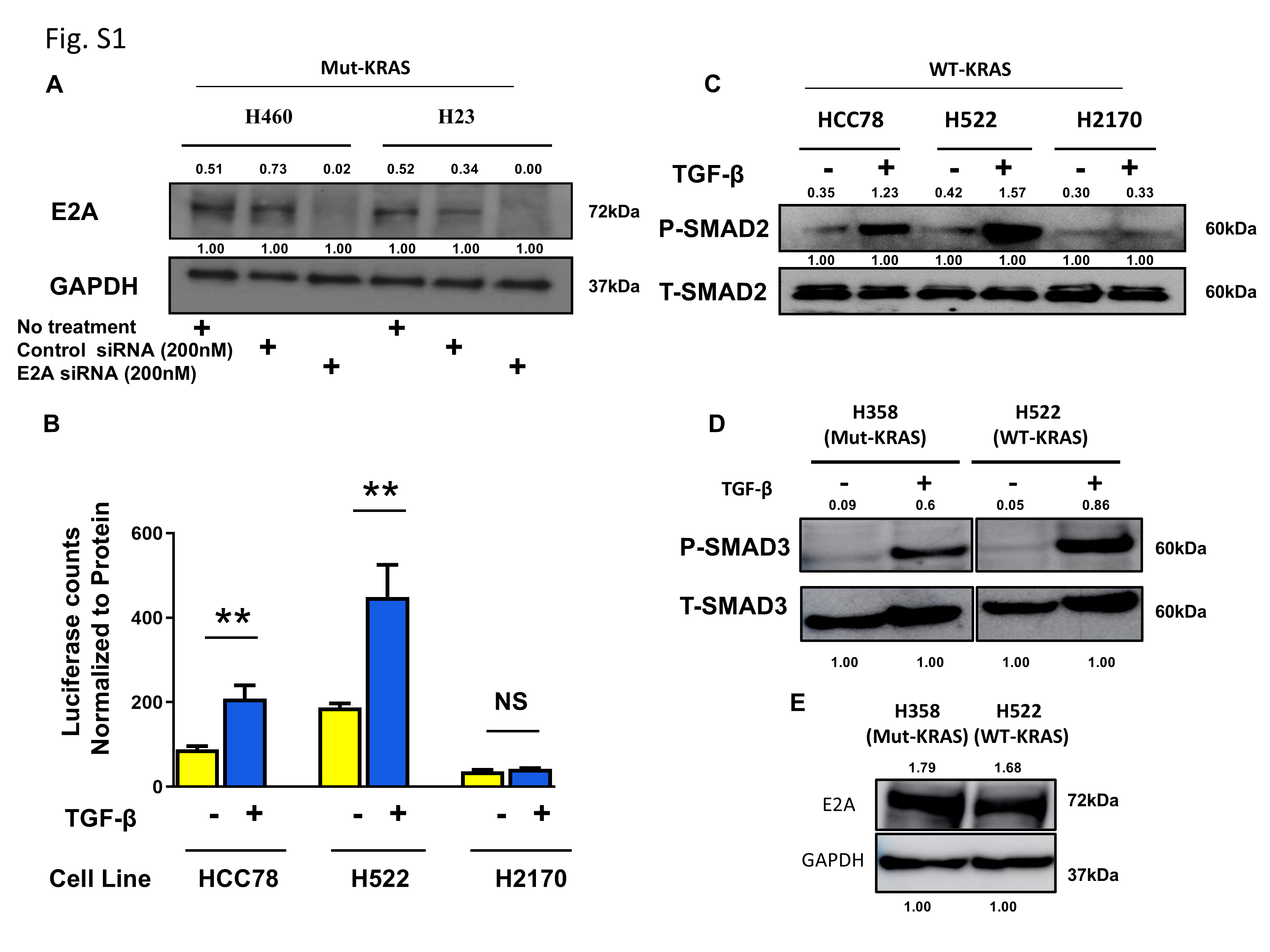

Supplement: Supplementary file 2 — Fig. S1. Validation of siRNA‐mediated knockdown of E2A in mutant KRAS cell lines and SMAD2 activation in wild‐type KRAS cell lines. [file MOL2-20-1877-s003.tif]

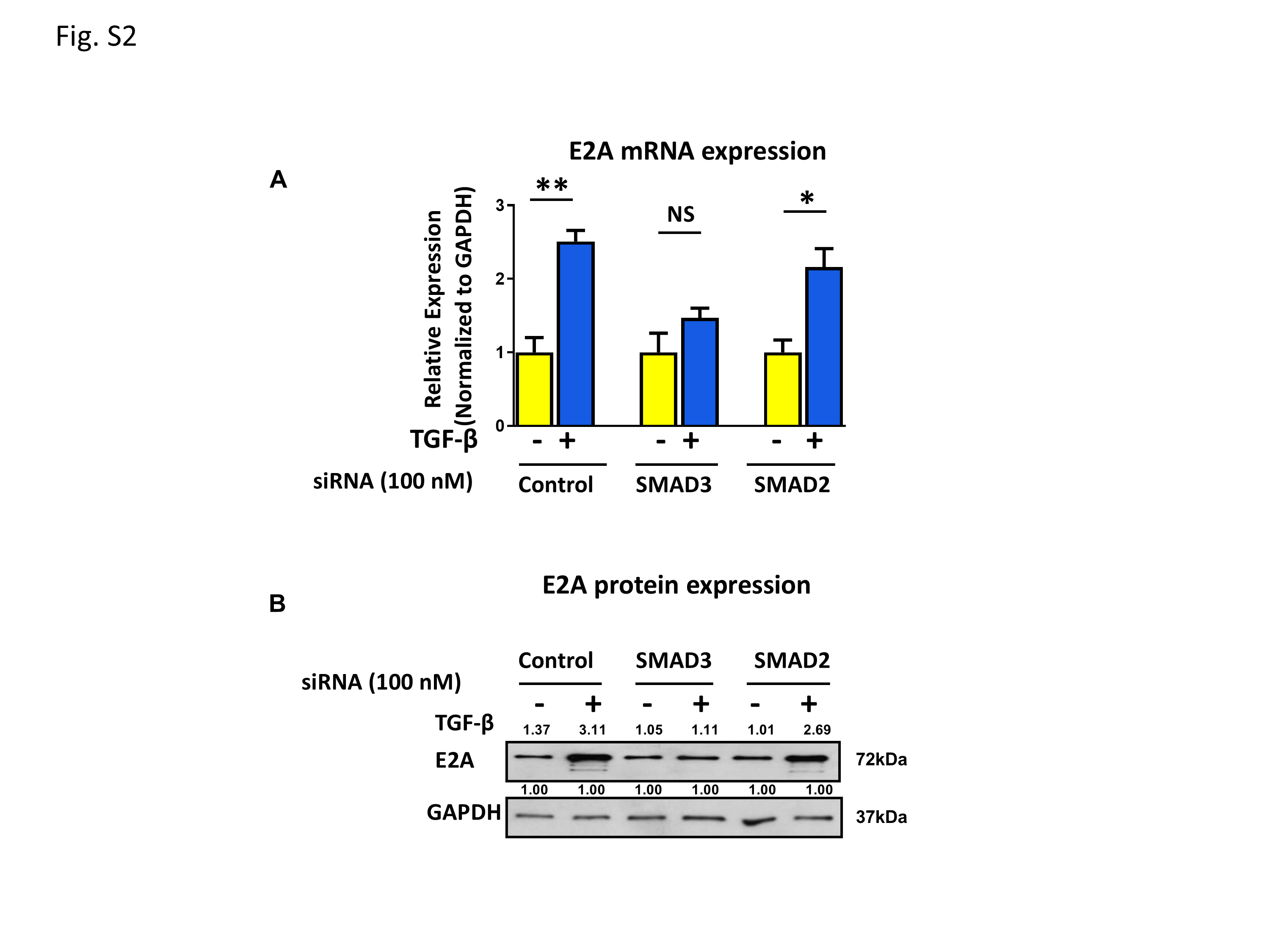

Supplement: Supplementary file 3 — Fig. S2. TGF‐β1 induces E2A expression in a SMAD3‐dependent manner. [file MOL2-20-1877-s006.tif]
