# Supplementary material for: E2A selectively regulates TGF‐β–induced apoptosis in KRAS‐mutant non‐small cell lung cancer
Source: Mol Oncol. 2026 Mar 17;20(7):1877–88. doi: 10.1002/1878-0261.70236 (PMC13352951; doi:10.1002/1878-0261.70236)
Supplement: Supplementary file 8 — Data S1. Supplementary Legends. [file MOL2-20-1877-s007.docx]

**Supplementary Information**

**Table 1. qPCR SuperArray analysis of apoptosis-related gene expression.**
Expression of apoptosis-regulating genes was assessed using a qPCR SuperArray. Fold changes were calculated according to the manufacturer’s protocol. Gene expression following TGF-β treatment was normalized to respective untreated controls.

**Figure S1. Validation of siRNA-mediated knockdown of E2A in mutant KRAS cell lines and SMAD2 activation in wild-type KRAS cell lines.**
(A) Mut-KRAS cell lines H460 and H23 cells were transfected with 200 nM control or E2A siRNA. E2A protein levels were assessed after 72 hours by Western blot. Band intensities were quantified and normalized to the corresponding GAPDH loading control. The normalized protein expression values are shown above each respective band.

(B) SMAD2/3 transcriptional activity was assessed using a SMAD-responsive luciferase reporter (CAGA-luc) transfected into HCC78, H2170, or H522 cells. Cells were serum-starved for 24 hours and treated with or without TGF-β1 (5 ng/mL). After 4 hours, luciferase activity was measured using the Dual-Luciferase Assay System (Promega).
(C) WT-KRAS HCC78, H522 and H2170 cells were treated with or without TGF-β1 (5 ng/mL) for 1 hour. Phospho-SMAD2 or total-SMAD2 levels were assessed by Western blot. Total-SMAD2 served as the loading control. Band intensities were quantified and normalized to the corresponding total-SMAD2 bands. The normalized protein expression values are shown above each respective band.

(D) Mut-KRAS H358 and WT-KRAS H522 cells were treated with or without TGF-β1 (5 ng/mL) for 1 hour. Phospho-SMAD3 or total-SMAD3 levels were assessed by Western blot. Total-SMAD3 served as the loading control. Band intensities were quantified and normalized to the corresponding total-SMAD3 bands. The normalized protein expression values are shown above each respective band.

(E) E2A protein level was assessed by Western blotting in Mut-KRAS H358 and WT-KRAS H522 cells. Band intensities were quantified and normalized to the corresponding GAPDH bands. The normalized protein expression values are shown above each respective band.

Statistical analysis was performed using a two-tailed unpaired Welch’s t-test. Bars represent mean ± SD, with n = 3 biological replicates. Statistical significance was defined as *NS*, not significant; ***P* < 0.01.

**Figure S2. TGF-β1 induces E2A expression in a SMAD3-dependent manner.**
(A) A549 cells were transfected with control, SMAD3, or SMAD2 siRNA and stimulated with TGF-β1 (5 ng/mL). E2A mRNA expression was assessed by qPCR after 72 hours.
(B) E2A protein levels were evaluated by Western blotting under the same conditions. GAPDH was used as a loading control. Band intensities were quantified and normalized to the corresponding GAPDH bands. The normalized protein expression values are shown above each respective band.

Statistical analysis was performed using a two-tailed unpaired Welch’s t-test. Bars represent mean ± SD, with n = 3 biological replicates. Statistical significance was defined as *NS*, not significant; **P* < 0.05; ***P* < 0.01.

**Supplementary video files: Time-lapse videos of caspase-3 activation in A549 cells treated with E2A or control siRNA with or without TGF-β1 treatment.**
A549 cells were transfected with control (1-Control-siRNA) or E2A (3-E2A-siRNA) siRNA and treated with TGF-β1 (5 ng/mL) for 72 hours (2- Control-siRNA-TGF and 4- E2A-siRNA-TGF). Caspase-3 activation was visualized by live-cell imaging using NucView 488 in an IncuCyte system.
